# Border Trap Characterizations of Al_2_O_3_/ZrO_2_ and Al_2_O_3_/HfO_2_ Bilayer Films Based on Ambient Post Metal Annealing and Constant Voltage Stress

**DOI:** 10.3390/nano10030527

**Published:** 2020-03-15

**Authors:** Md. Mamunur Rahman, Dae-Hyun Kim, Tae-Woo Kim

**Affiliations:** 1 School of Science and Engineering, Department of EEE, Canadian University of Bangladesh, Dhaka 1213, Bangladesh; rahman.mamun37@gmail.com; 2 School of Electrical Engineering, University of Ulsan, Ulsan 44610, Korea; 3 School of Electronics Engineering, Kyungpook National University, Daegu 702-701, Korea

**Keywords:** III–V semiconductor, atomic layer deposition, border trap, constant voltage stress, high-*k*, interface trap, post metal annealing

## Abstract

This study represents a comparison of the border trap behavior and reliability between HfO_2_ and ZrO_2_ films on *n*-In_0.53_Ga_0.47_As with an Al_2_O_3_ interfacial layer. The effect of different post metal annealing conditions on the trap response was analyzed and it was found that the N_2_:H_2_ mixed FGA passivates the border trap quite well, whereas N_2_-based RTA performs better on interface traps. Al_2_O_3_/HfO_2_ showed more degradation in terms of the threshold voltage shift while Al_2_O_3_/ZrO_2_ showed higher leakage current behavior. Moreover, Al_2_O_3_/ZrO_2_ showed a higher permittivity, hysteresis, and breakdown field than Al_2_O_3_/HfO_2_.

## 1. Introduction

As potential gate insulator candidates in III–V channel material-based nano-metric metal oxide semiconductor field-effect transistors (MOSFETs), which are considered as a future device for logic applications with a higher speed and bottled-up power consumption, the most studied Hf and Zr-based high-*k* oxides suffer from a lower barrier elevation as well as a destitute interface with the semiconductor material compared to the SiO_2_/Si-based system. These shortcomings are hindrances to achieving the leakage current challenge [1,2,3,4,5]. To solve these issues, an interfacial layer of Al_2_O_3_ is added between the above-mentioned dielectric materials and semiconductor. This forms a bilayer arrangement of a gate oxide structure since Al_2_O_3_ possesses the supremacy of a higher bandgap with a significant barrier offset and an improved surface passivation scheme with the channel material [5,6,7]. 

Among the III–V family, which is considered as a next-generation channel material instead of Si as it is in the material limit, indium-rich In*_x_*Ga_1-*x*_As materials with *x* = 0.53 have received a lot of attention due to their nearly eight times higher electron mobility compared to Si and their higher injection velocity. In addition, these materials have already been developed in defense and high-frequency analog applications [8,9]. The high velocities are attained by reimbursing a lower effective mass which causes the “density of state bottleneck” dilemma which pins the fermi level, E_F_, inside the conduction band, resulting in a reduction of the conduction band distinction height [9,10,11]. This disposition of the fermi level makes itself align with the border trap’s energy levels, which are located near the interfacial oxide region with the semiconductor inside the oxide [10,12]. When an AC signal is superimposed with the applied DC bias, there is a tunneling of channel electrons into or emitting between the border traps and the semiconductor. Usually, these near interfacial traps are categorized by their position inside the oxide; the furthermost trap takes the longest time to fill. So this charge exchange time is characterized by the depth of the traps inside the oxide which also depends on the applied frequency [10,13]. This creates a frequency dependent capacitance response in the accumulation and these traps are also responsible for dilapidation of mobility, on-state current, transconductance, and reliability by causing high hysteresis, threshold voltage instability, and phonon scattering [10,11,12,14]. Moreover, as a reliability issue, it has already been reported that the constant-voltage-stress (CVS) is responsible for electron trapping in these acceptors like oxide traps as well as the creation of new oxygen vacancy defects [8,15,16].

The conventional interface trap model is unable to explain the border trap behavior due to a time constant mismatch between both types of traps, as well as the border trap estimation from capacitance-voltage (C–V) hysteresis, which suffers from complete re-emission of captured charge at the C–V reverse sweep. Consequently, it is appropriate to characterize these traps by regarding accumulation frequency dispersion [3,17]. Furthermore, there are already several reports regarding border trap reduction by following some annealing process, although a clear understanding of the annealing ambient is lacking [16,17,18]. In addition, although both HfO_2_ and ZrO_2_ are considered to have almost the same electrical and chemical properties and there are reports of their physical, chemical, and electrical characterizations, there is still an opportunity to investigate the oxide trap characterization between these oxides [6,16]. In this study, we characterized the trap responses between HfO_2_ and ZrO_2_ oxides along with an Al_2_O_3_ interfacial layer in a bilayer form with different annealing environments as well as under different stress voltage conditions in the CVS environment.

## 2. Materials and Methods 

The Al_2_O_3_, HfO_2_, and ZrO_2_ films were deposited on *n*-In_0.53_Ga_0.47_As by atomic layer deposition (ALD) using trimethylaluminum (TMA), tetrakis (ethymethylamino) hafnium (TEMAH), and ZrCl_4_ as the metal precursors for Al_2_O_3_, HfO_2_, and ZrO_2_, respectively, where H_2_O was the oxidant and N_2_ was used as both the carrier and purge gas. The details of the epitaxial growth of n-In_0.53_Ga_0.47_As on a 300 mm thick *n*-Si (001) substrate were described in our previous report [19]. Before deposition, the substrate was cleaned by a standard wet cleaning process, which incorporated hydrochloric acid (HCl) and deionized (DI) water to remove the contaminants and native oxide from the surface. Then, the substrate was dried in a nitrogen(N_2_) environment for the prevention of water mask formation on the surface and transferred to the ALD chamber (“Atomic Classic”, CN1, Gyenggi-do, Korea) within a minimal time interval. Before the actual film deposition, the substrates were pretreated with 10 cycles of TMA pulses to passivate the surface due to its’ “self-cleaning effect” [10]. Then, two individual depositions of Al_2_O_3_/ZrO_2_ (1 nm/3.3 nm) and Al_2_O_3_/HfO_2_ (1 nm/3 nm) were performed followed by ALD TiN (5 nm) deposition on the top of the oxide layer. Then, for the front side metal electrode, a layer of Ti/Au (200/2000 Å) was deposited by e-beam evaporation (Temescal, Zeus Co, Ltd.; Yongin, Korea, model: FC-2000) via lift-off and the same metal layer was also deposited for the backside contact. To isolate the metal-oxide-semiconductor capacitors (MOSCAPS), reactive ion etching (RIE) was performed based on SF_6_/Ar gas (30/10 sccm) to remove the TiN layer. Then, the devices were separately processed by post-metal annealing (PMA) at 350 °C in a N_2_, H_2_, and O_2_ environment for 1 min to observe the passivation effect on the electrically active defects in the high-*k*/In_0.53_Ga_0.47_As interface and oxide itself. Another set of devices were annealed in forming gas (N_2_:H_2_ = 96%:4%) for 30 min at 300 °C. The electrical characterizations were carried out in the dark environment using a Keithley 4200A-SCS parameter analyzer (Tektronix, Inc., Beaverton, OR, USA) at room temperature and the CVS measurements were obtained using a Keysight CV-enabled B1500A semiconductor device parameter analyzer. 

## 3. Results and Discussion

**Figure 1:**
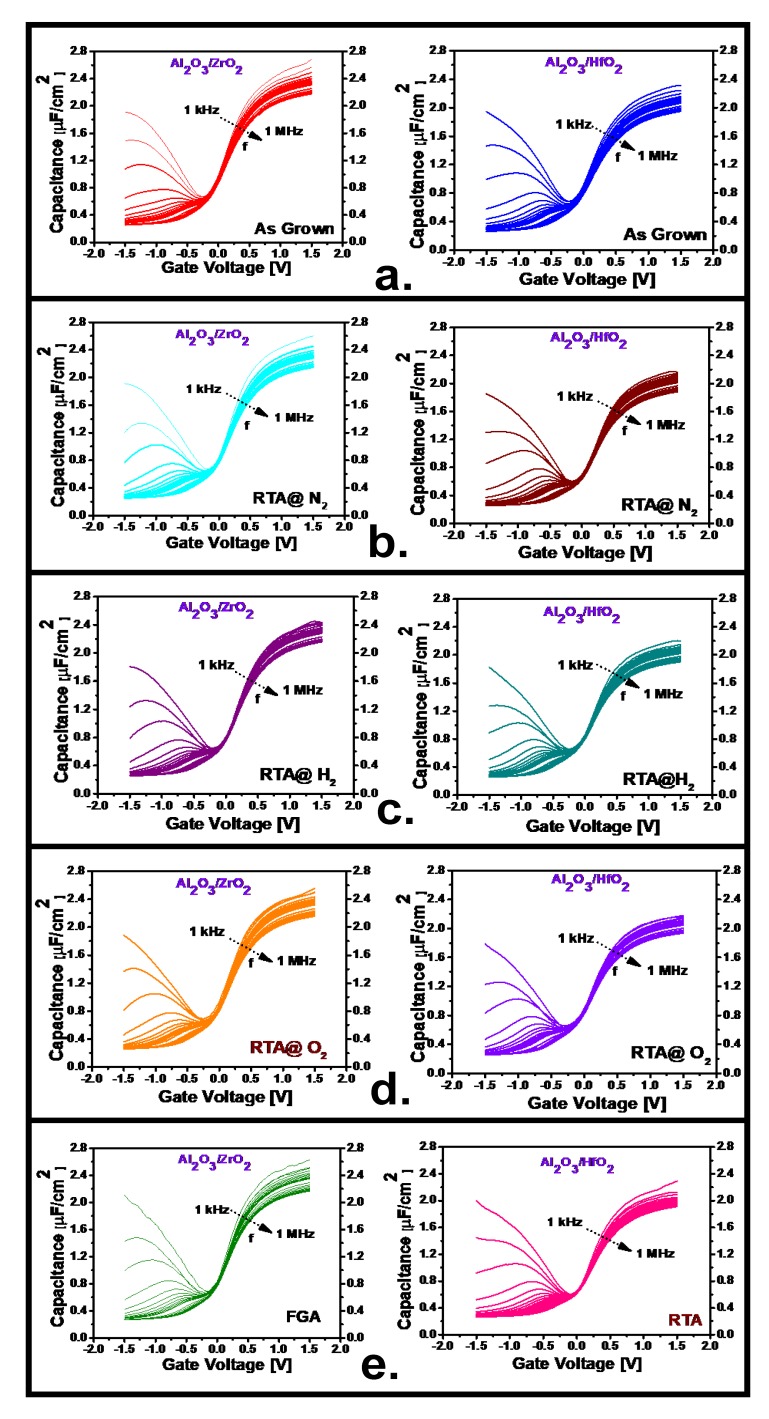

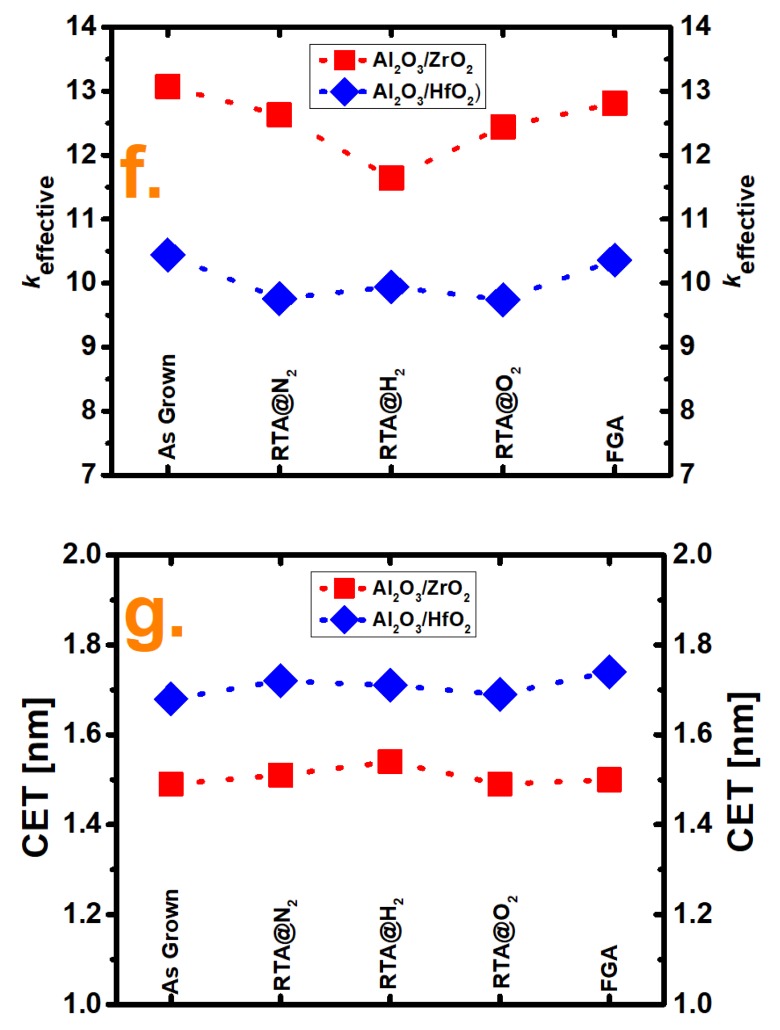
(**a**–**e**) Frequency dispersion (1 kHz–1 MHz) capacitance voltage (C–V) response of Al_2_O_3_/ZrO_2_ and Al_2_O_3_/HfO_2_ respectively, for as-grown and different post-metal annealing (PMA) treatment conditions, at applied gate voltages ranging from −1.5 to +1.5 V. (**f**) Effective dielectric constant (*k*_effective_) and (**g**) capacitance equivalent thickness (CET) comparison of both samples under as-grown conditions and after different PMA treatments.

Figure 1 illustrates the measured capacitive-voltage response of the two samples along with the extracted dielectric constant (*k*_effective_) and the calculated capacitive equivalent thickness (CET). In Figure 1a, the measured frequency dependent C–V responses are plotted for 1 kHz–1 MHz with a voltage range of −1.5 V to +1.5 V for both samples under as-grown conditions. Although the inversion responses are the same in both cases, there is more dispersion in the accumulation region of the Al_2_O_3_/ZrO_2_ than in the Al_2_O_3_/HfO_2_, which indicates a higher density of border traps (N_bt_) presented in it. Furthermore, the higher accumulation capacitance also indicates greater permittivity of the Al_2_O_3_/ZrO_2_ film. From Figure 1b–e, frequency dispersion is presented of both samples for the cases of PMA treatments at different ambient. From all of these figures, it is evident that the frequency dispersion was reduced compared to the as-grown condition after these treatments although the amount of reduction varied based on the ambient type. This reduction indicates a minimization of border trap density (N_bt_) and the highest amount of trap depreciation was obliged for both samples by FGA treatment, which was indicated by the lowest amount of frequency dispersion as observed in Figure 1e. The amount of frequency dispersion along with border trap density (N_bt_) reductions are characterized later. Figure 1f demonstrates the extracted *k*_effective_ value from the measured 1 kHz frequency response of the two deposition cases depending on the different annealing treatments by using the process as described in our previous report [10]. The extracted *k*_effective_ values at the as-grown condition for the Al_2_O_3_/ZrO_2_ film are 13.07 and 10.44 for Al_2_O_3_/HfO_2_ while the permittivity values for Al_2_O_3_/ZrO_2_ in all PMA treatment cases are higher than those of Al_2_O_3_/HfO_2_, which indicates the higher permittivity of the ZrO_2_ film compared to HfO_2_ since Al_2_O_3_ has the same thickness in both cases [20]. Furthermore, the permittivity decreased in both samples after all types of PMA treatment compared to the as-grown condition, which indicates interfacial layer formation with a lower permittivity as well as some intermixing effect in between the high-*k* and InGaAs surface [20]. For the Al_2_O_3_/ZrO_2_ sample, the lowest permittivity value was found for the H_2_ treated case which was 11.64 and for the Al_2_O_3_/HfO_2_ sample, it was for the O_2_ treated case which had a value of 9.74. The other treated cases have the values within these limits. The CET values, as depicted in Figure 1g, extracted from the accumulation capacitance from 100 kHz at the maximum bias voltage, as mentioned in a previous report, for both samples have almost identical for both the as-grown and annealed conditions [10]. Although there is a little variation in CET values between different annealed conditions of both samples, from the figure, it can be inferred that the CET values of the as-grown samples had not faced a significant change. 

**Figure 2:**
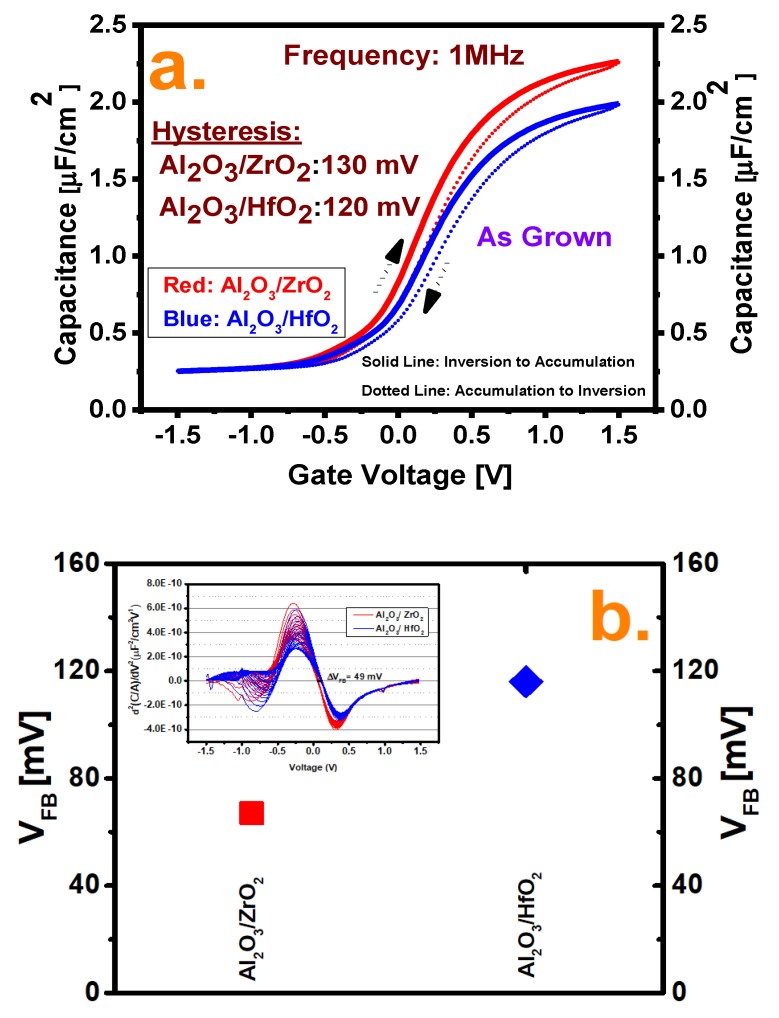
(**a**) Hysteresis comparison from −1.5 V to +1.5 V at 1 MHz for both cases. (**b**) Flat band voltage comparison for both films, as calculated by the inflection point method. Inset: Second derive of normalized C–V data for calculating the flat voltage shift.

Figure 2a shows the hysteresis comparison of the two samples under as-grown conditions measured at a frequency of 1 MHz to minimize the trap response by starting the C–V sweep at a sweep speed of 20 mV/s from inversion to accumulation and without any holding delay back to inversion. From the figure, it is detected that the Al_2_O_3_/ZrO_2_ sample shows higher hysteresis (130 mV) than Al_2_O_3_/HfO_2_ (120 mV). The higher hysteresis value indicates more charge trapping at the border traps. The charge traps into these vacancies when the fermi level becomes aligned with the trap energy level at the accumulation region and when the C–V sweep reverses back, which cannot be moved away unless the fermi level becomes closer to the valance band and makes a voltage shift. Figure 2b shows the flat-band voltages (V_FB_) of the two samples extracted by the infection point method by calculating the second derivative of normalized C–V data as illustrated in the inset of Figure 2b, where V_FB_ shows a left shift for Al_2_O_3_/ZrO_2_ compared to Al_2_O_3_/HfO_2_, which can be explained by the elimination of electron traps by the ZrO_2_ dielectric itself, as well as the incidence of positive charges [20,21].

**Figure 3:**
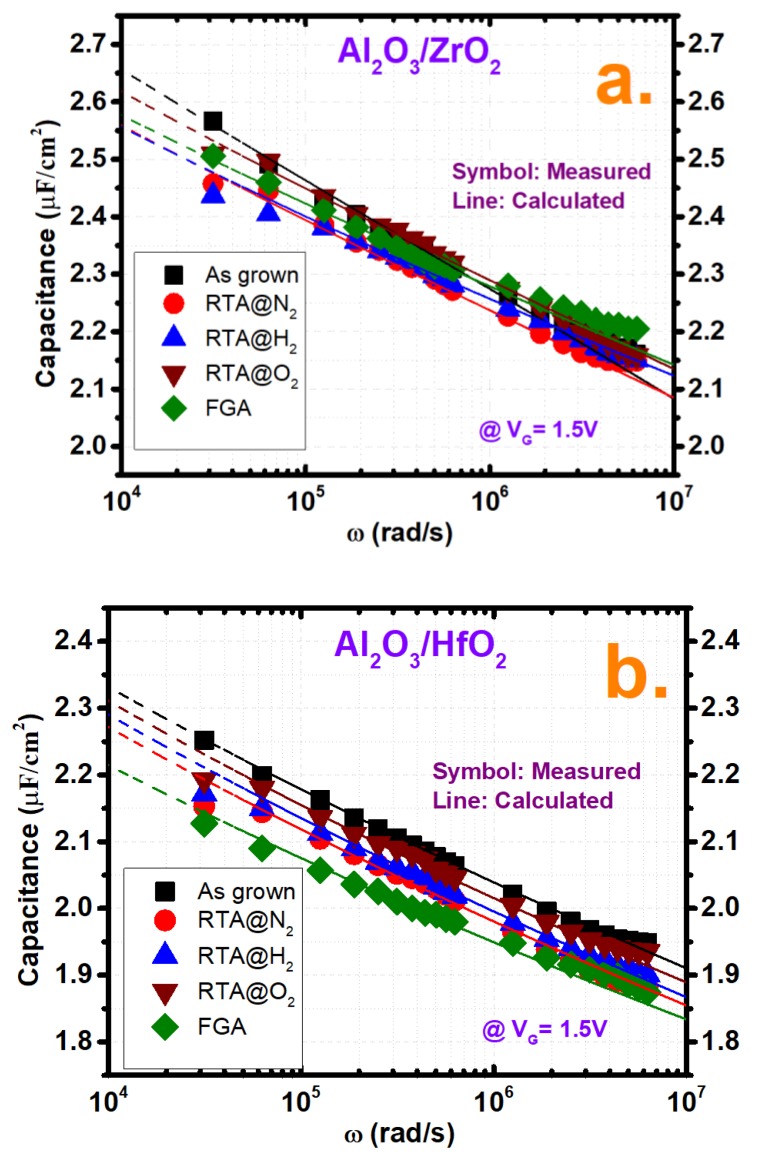

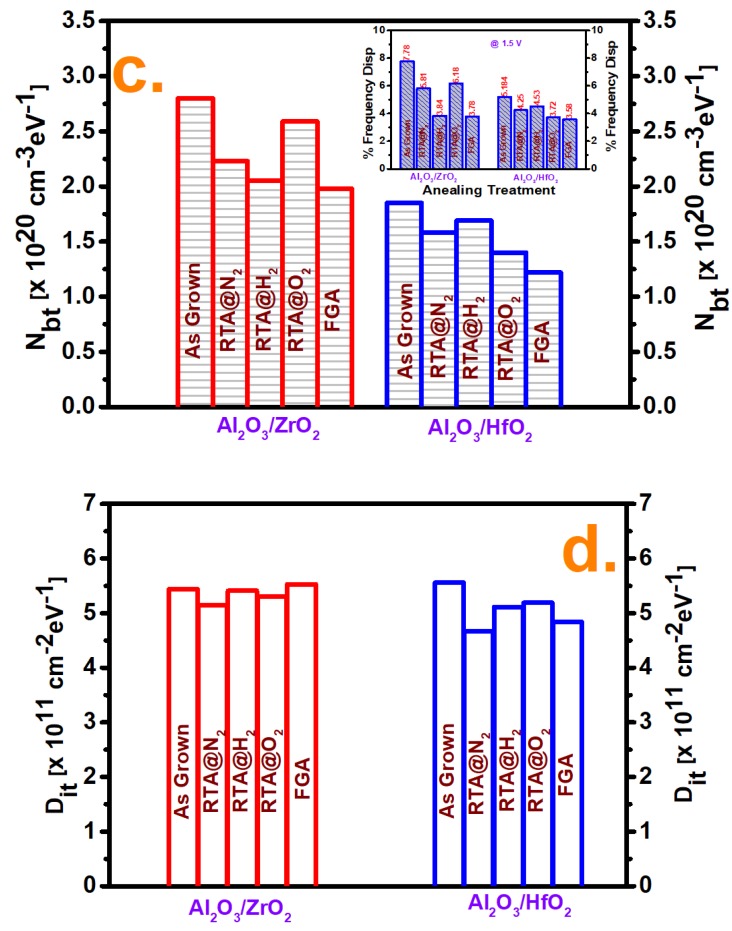
Fitted curves of the measured (symbols) and calculated (lines) capacitance values from the BT Distributed Border Trap model for both films for all annealing conditions including as-grown at 1.5 V for (**a**) Al_2_O_3_/ZrO_2_ and (**b**) Al_2_O_3_/HfO_2_. (**c**) Border trap density (N_bt_) and (**d**) interface trap density (D_it_) comparison of both films for all annealing conditions including as-grown. The inset in (**c**) shows the frequency dispersion comparison for the above-mentioned criteria.

Figure 3 depicts the trap characterizations as well as the frequency dispersions of both samples under different annealing conditions. The border trap density (N_bt_) was characterized by the distributed border trap model proposed by Yaun et al. by making the best fit between the measured capacitance at the specific voltage in the accumulation region and the capacitance calculated from the model [22]. In this model, the total oxide thickness is segmented into a small number of quantities. Every quantity represents a certain amount of oxide capacitance which is in a parallel configuration of admittance that is proportional to border trap quantities and is in a series configuration with semiconductor capacitance. A detailed explanation of this model and extraction process of N_bt_ was described in our previous report [10]. However, in the extraction process, the effective electron masses of the Al_2_O_3_, HfO_2_, and ZrO_2_ films were considered as 0.23 m_0_, 0.22 m_0_, and 0.3 m_0_, respectively, where m_0_ represents the electron rest mass [17,23]. In addition, a one-dimensional Poisson–Schrodinger solver simulation tool (Nextnano) was used to calculate the semiconductor capacitance C_s_ at border trap extraction voltage [24]. Figure 3a, b shows the fitting curves between the measured and calculated capacitance for both cases. From Figure 3c, it is observed that N_bt_ is higher in the Al_2_O_3_/ZrO_2_ (2.8 × 10^20^ cm^−3^·eV^−1^) film compared to the Al_2_O_3_/HfO_2_ (1.85 × 10^20^ cm^−3^·eV^−1^) film as more frequency dispersion is observed in the Al_2_O_3_/ZrO_2_ film earlier. The extracted N_bt_ values for the Al_2_O_3_/ZrO_2_ sample after PMA treatment were, 2.23 × 10^20^ cm^−3^·eV^−1^, 2.05 × 10^20^ cm^−3^·eV^−1^, 2.59 × 10^20^ cm^−3^·eV^−1^ and 1.98 × 10^20^ cm^−3^·eV^−1^ at N_2_, H_2_, O_2_ and FGA annealing cases, respectively, while on the other hand for Al_2_O_3_/HfO_2_ samples, the values were 1.58 × 10^20^ cm^−3^·eV^−1^, 1.69 ×10^20^ cm^−3^·eV^−1^, 1.4 × 10^20^ cm^−3^·eV^−1^ and 1.22 × 10^20^ cm^−3^·eV^−1^ at N_2_, H_2_, O_2_ and FGA annealing cases, respectively. So, as depicted, the N_bt_ values show a decrease after different annealing treatments, where values are lower with the fully H_2_ ambient-based treatment and at the lowest level with the FGA treatment, which involves a combination of H_2_ and N_2_ ambient in both samples. Therefore, it is evident that the H_2_-based heat treatment was quite effective in reducing acceptor-like electron traps, which was also reported by Jun Lin et al. [17]. The frequency dispersion shown in the inset of Figure 3c, which was calculated as described in a previous report, shows a similar trend as the border traps since the dispersion is mainly originated due to these traps [7]. The measured frequency dispersions for the as grown condition of Al_2_O_3_/ZrO_2_ and Al_2_O_3_/HfO_2_ samples were 7.78% and 6.184% respectively, while the lowest values were found for FGA cases which are 3.78% and 3.68%, respectively. The interface trap density (D_it_) of the two samples, which is calculated by the conductance method by considering the series resistance correction, is illustrated in Figure 3d under different treatments along with the as-grown sample [25]. The D_it_ values of the Al_2_O_3_/ZrO_2_ and Al_2_O_3_/HfO_2_ samples at the as-grown conditions were almost identical with values of 5.44 × 10^11^ cm^−2^·eV^−1^ and 5.56 × 10^11^ cm^−2^·eV^−1^, respectively, since both samples had the same interface, identical Al_2_O_3_ layer thicknesses, and the same pre-treatment. Additionally, the annealing treatment using N_2_ ambient showed the highest reduction of D_it_ in both samples compared to the other environment, where the reduced values were 5.14 × 10^11^ cm^−2^·eV^−1^ and 4.67 × 10^11^ cm^−2^·eV^−1^ for Al_2_O_3_/ZrO_2_ and Al_2_O_3_/HfO_2_ cases, respectively.

**Figure 4:**
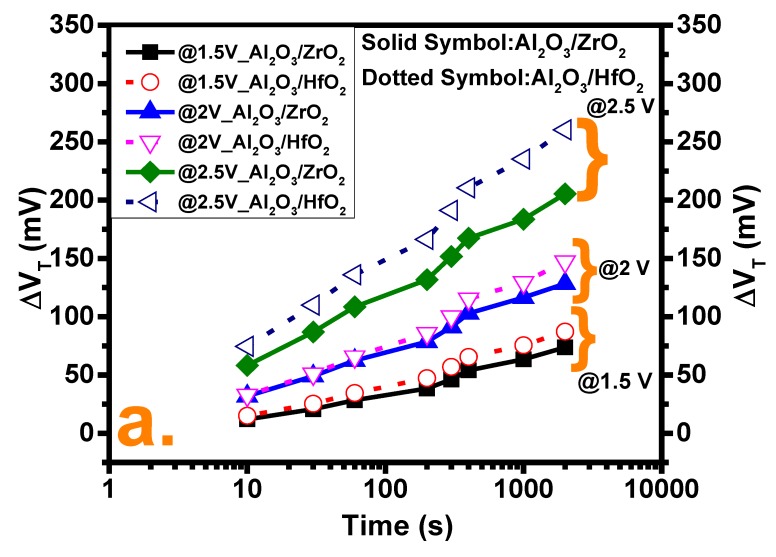

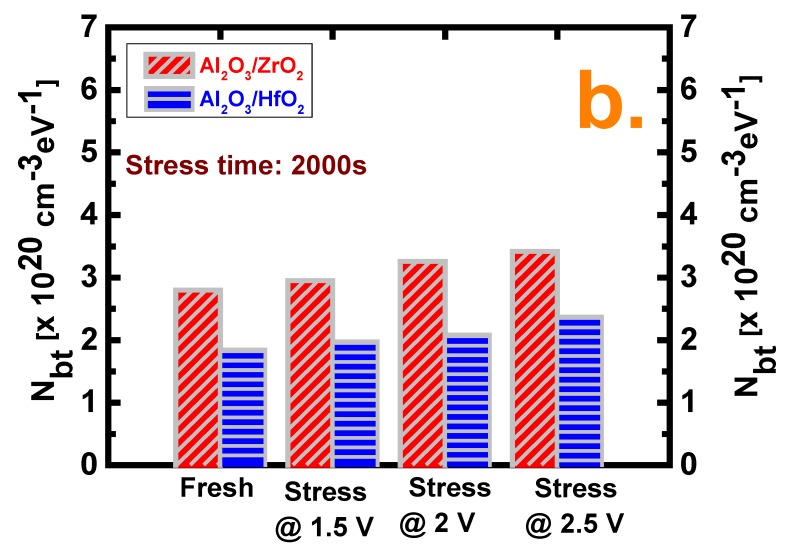
(**a**) Threshold voltage shift (V_TH_) after constant voltage stress (CVS) at three different voltages for both samples. (**b**) Border trap density (N_bt_) characterizations of both cases after CVS including fresh samples.

The reliability of the as-grown samples was checked by CVS at three different bias conditions, 1.5 V, 2 V, and 2.5 V, for a time frame of 1000 s where the stress was intermittent after some explicit time frame to allow the C–V measurement to calculate the threshold voltage shift (V_TH_). From Figure 4a, it is evident that V_TH_ shows a positive shift at positive bias stress, which indicates electron trapping from the semiconductor to traps in the oxide and the passivation of positive charge where the Al_2_O_3_/HfO_2_ sample shows a greater shift in all three cases [26]. The lower V_TH_ degradation of the Al_2_O_3_/ZrO_2_ film can be explained by the grain morphology of the oxide film. Meanwhile, it is assumed that oxygen straightforwardly diffuses through the grain boundaries to passivate the oxygen vacancies at grain margins or inside of them. Since the ZrO_2_ film has a smaller and more uniform grain orientation, it makes the diffusion of oxygen into the grain or regions near it easier, which eventually reduces the oxygen vacancy concentration [16]. The N_bt_ characterization after a different stress bias at 1000 s is demonstrated in Figure 4b, which depicts a linear relationship with the traps compared with the fresh sample. The increase of N_bt_ with a more positive bias can be explained considering that the larger bias pushes the E_F_ deeper into the conduction band. This results in a larger electric field across the oxide, E_ox_, so that more border traps can be assessed since these traps are distributed at diverse energy levels and also several depths into the oxide [27]. 

**Figure 5:**
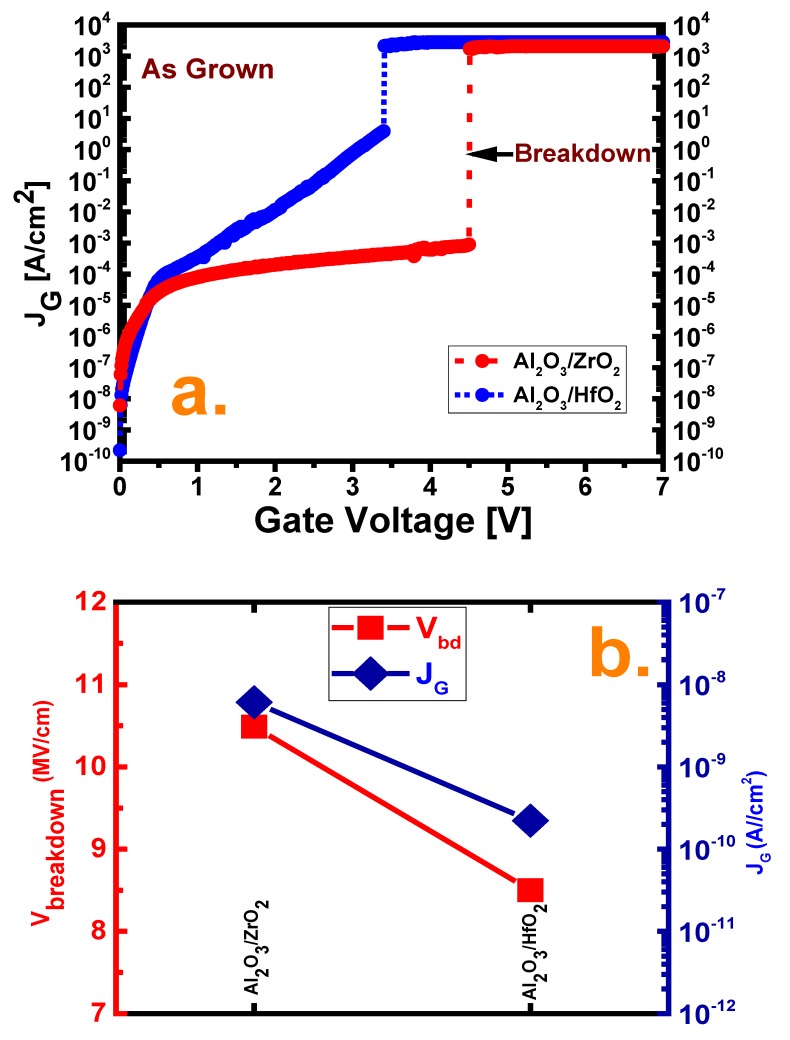
(**a**) Leakage current-voltage (J–V) profile under a positive gate voltage and (**b**) breakdown voltage (V_BD_) and leakage current density (J_G_) comparison for all deposition cases.

Figure 5 depicts the measured current-voltage (J_G_–V) characteristics along with the breakdown voltages of the two samples. The higher leakage current of the Al_2_O_3_/ZrO_2_ film may be attributed to the lower conduction band offset of the ZrO_2_ film compared with the HfO_2_ film as well as higher number of traps in ZrO_2_ as depicted earlier [16,20]. This lower band offset may be attributed to a greater leakage of electron flow which was further assisted by the existing traps. Moreover, assisted tunneling with the rapidly increased leakage current for Al_2_O_3_/HfO_2_ may be a result of direct tunneling conduction [9,16,28]. However, further investigation is needed to clarify this hypothesis. The higher breakdown voltage of the Al_2_O_3_/ZrO_2_ film, i.e., 10.49 MV/cm higher than the Al_2_O_3_/HfO_2_ film (8.5 MV/cm), may be attributed to the uniform grain orientation of the ZrO_2_ film, as mentioned earlier as well as thermal issues at the time of processing [28].

## 4. Conclusions

In conclusion, between the bilayers, Al_2_O_3_/ZrO_2_ shows higher permittivity and accumulation dispersion compared to Al_2_O_3_/HfO_2_ while Al_2_O_3_/HfO_2_ shows more degradation in terms of reliability. The larger frequency dispersion can be attributed to the higher N_bt_ while the larger V_TH_ is due to nonuniformity of the grain size. The frequency dispersion showed a reduction after different types of annealing, which corresponds to a reduction of N_bt_ where FGA resulted in the best passivation. Although D_it_ shows similar behavior in both samples, the leakage current is higher in the Al_2_O_3_/ZrO_2_ film due to the lower band offset.
